# Supplementary material for: Endurance Runners with Intramyocellular Lipid Accumulation and High Insulin Sensitivity Have Enhanced Expression of Genes Related to Lipid Metabolism in Muscle
Source: J Clin Med. 2020 Dec 6;9(12):3951. doi: 10.3390/jcm9123951 (PMC7762159; doi:10.3390/jcm9123951)
Supplement: Supplementary file 1 [file jcm-09-03951-s001.pdf]

Supplementary Table S1. Upregulated genes differentially expressed in athletes ($p < 0.05$ and 1.3-fold change)

	Gene Symbol	*p* Value	Fold Change
221051_s_at	*NMRK2*	0.01	2.38
236518_at	*CCDC183*	0.00	2.05
218804_at	*ANO1*	0.00	2.05
234675_x_at		0.01	2.02
207076_s_at	*ASS1*	0.00	1.85
209135_at	*ASPH*	0.02	1.81
228434_at	*BTNL9*	0.03	1.81
229985_at	*BTNL9*	0.01	1.79
215795_at	*MYH7B*	0.01	1.78
217979_at	*TSPAN13*	0.01	1.77
230992_at	*BTNL9*	0.01	1.75
226884_at	*LRRN1*	0.03	1.74
220039_s_at	*CDKAL1*	0.01	1.73
236520_at		0.02	1.72
219895_at	*TMEM255A*	0.04	1.72
201030_x_at	*LDHB*	0.00	1.69
233824_at		0.00	1.69
232257_s_at		0.05	1.67
236359_at	*SCN4B*	0.04	1.64
242868_at		0.00	1.63
1557286_at		0.01	1.63
202780_at	*OXCT1*	0.01	1.63
1556542_a_at		0.04	1.63
209992_at	*PFKFB2*	0.04	1.63
205247_at	*NOTCH4*	0.01	1.62
1554182_at	*TRIM73///TRIM74*	0.00	1.61
232892_at	*MIR1-1HG*	0.02	1.61
204726_at	*CDH13*	0.01	1.6
1561167_at		0.01	1.6
1565821_at		0.01	1.6
210169_at	*SEC14L5*	0.01	1.6
236963_at		0.02	1.6
1552880_at	*SEC16B*	0.02	1.6
235228_at	*CCDC85A*	0.02	1.6
1568623_a_at	*SLC35E4*	0.00	1.59
204844_at	*ENPEP*	0.00	1.59
1552256_a_at	*SCARB1*	0.02	1.59
1557283_a_at	*ZNF519*	0.02	1.59
1557293_at	*LINC00969*	0.03	1.59
231644_at		0.01	1.58
228115_at	*GAREM1*	0.01	1.58
223687_s_at	*LY6K*	0.02	1.58
231779_at	*IRAK2*	0.03	1.58
243332_at	*LOC105379610*	0.04	1.58
232118_at		0.01	1.57
203423_at	*RBP1*	0.02	1.57
208498_s_at	*AMY1A///AMY1B///AMY1C///AMY2A///AMY2B///AMYP1*	0.03	1.57
237154_at	*LOC101930114*	0.00	1.56
1559691_at		0.01	1.56

243481_at	*RHOJ*	0.03	1.56
238834_at	*MYLK3*	0.01	1.55
213438_at	*NFASC*	0.02	1.55
242290_at	*TACC1*	0.04	1.55
1570255_s_at	*ANKRD20A1///ANKRD20A12P///ANKRD20A2///ANKRD20A3///ANKRD20A4///ANKRD20A8P///LOC102723552*	0.05	1.55
238752_at	*GPLD1*	0.02	1.54
227100_at	*B3GLCT*	0.05	1.54
203818_s_at	*SF3A3*	0.05	1.54
222379_at	*KCNE4*	0.00	1.53
222802_at	*EDN1*	0.01	1.53
213564_x_at	*LDHB*	0.00	1.52
239379_at		0.01	1.52
1556156_at	*ESRRB*	0.01	1.52
202499_s_at	*SLC2A3*	0.01	1.52
225847_at	*NCEH1*	0.02	1.52
209710_at	*GATA2*	0.00	1.51
213715_s_at	*KANK3*	0.00	1.51
215555_at		0.03	1.51
239035_at	*MTHFR*	0.03	1.51
1553961_s_at	*SNX21*	0.01	1.5
206481_s_at	*LDB2*	0.02	1.5
227183_at	*CARMN*	0.03	1.5
209155_s_at	*NT5C2*	0.02	1.49
205911_at	*PTH1R*	0.02	1.49
207063_at	*TTTY14*	0.03	1.49
1554250_s_at	*TRIM73*	0.04	1.49
213316_at	*KIAA1462*	0.00	1.48
205304_s_at	*KCNJ8*	0.00	1.48
205303_at	*KCNJ8*	0.01	1.48
233949_s_at	*MYH7B*	0.02	1.48
236905_at	*NUP133*	0.02	1.48
223172_s_at	*MTFP1*	0.05	1.48
239660_at	*RALGAPA2*	0	1.47
228301_x_at	*NDUFB10*	0.01	1.47
200884_at	*CKB*	0.02	1.47
231923_at	*TMEM150C*	0.04	1.47
226908_at	*LRIG3*	0.01	1.46
207545_s_at	*LOC101928143///NUMB*	0.02	1.46
215375_x_at	*LRRFIP1*	0.02	1.46
205250_s_at	*CEP290*	0.03	1.46
205887_x_at	*MSH3*	0.03	1.46
236313_at	*CDKN2B*	0.05	1.46
212553_at	*RPRD2*	0.00	1.45
213206_at	*GOSR2*	0.01	1.45
218479_s_at	*XPO4*	0.01	1.45
206144_at	*MAGI1*	0.01	1.45
1553613_s_at	*FOXC1*	0.02	1.45
1557270_at		0.02	1.45
1558748_at		0.03	1.45
1554769_at	*ZNF785*	0.04	1.45
217506_at		0.02	1.44
219970_at	*GIPC2*	0.02	1.44

233197_at	*KLHL9*	0.03	1.44
226334_s_at	*AHSA2*	0.03	1.44
220961_s_at	*TBRG4*	0.03	1.44
215299_x_at	*SULT1A1*	0.03	1.44
225474_at	*MAGI1*	0.01	1.43
229377_at	*GRTP1*	0.01	1.43
236178_at	*LINC01590///SMIM8*	0.01	1.43
65630_at	*TMEM80*	0.02	1.43
243158_at		0.03	1.43
206188_at	*ZNF623*	0.03	1.43
210096_at	*CYP4B1*	0.03	1.43
212203_x_at	*IFITM3*	0.03	1.43
1556222_at	*SEPT7P9*	0.04	1.43
1569542_at	*ADGRA3*	0.05	1.43
55872_at	*UCKL1///ZNF512B*	0.00	1.42
223006_s_at	*TMEM245*	0.00	1.42
34225_at	*NELFA*	0.02	1.42
227289_at	*PCDH17*	0.03	1.42
1559566_at	*FBXO42*	0.04	1.42
213241_at	*PLXNC1*	0.04	1.42
232002_at		0.01	1.41
1557195_at		0.01	1.41
219165_at	*PDLIM2*	0.02	1.41
218476_at	*POMT1*	0.02	1.41
227334_at	*USP54*	0.03	1.41
1557383_a_at	*LOC105376896*	0.03	1.41
227379_at	*MBOAT1*	0.03	1.41
218700_s_at	*RAB29*	0.03	1.41
223619_x_at	*PECR*	0.04	1.41
230077_at	*LOC220729///SDHA///SDHAP1///SDHAP2*	0.04	1.41
1560031_at	*FRMD4A*	0.04	1.41
232297_at	*KLHL5*	0.04	1.41
1559425_at		0.04	1.41
229768_at	*OR51E1*	0.04	1.41
236072_at		0.00	1.4
1558256_at	*LINC00662*	0.00	1.4
226497_s_at	*FLT1*	0.01	1.4
236437_at		0.01	1.4
228776_at	*GJC1*	0.01	1.4
212230_at	*PLPP3*	0.02	1.4
1553960_at	*SNX21*	0.02	1.4
244045_at		0.02	1.4
228150_at	*SEC16B*	0.02	1.4
230061_at	*TM4SF18*	0.02	1.4
239694_at	*TRIM7*	0.02	1.4
228977_at	*LOC729680*	0.03	1.4
207981_s_at	*ESRRG*	0.03	1.4
232601_at		0.03	1.4
224685_at	*MLLT4*	0.03	1.4
215172_at	*LOC105369264///PTPN20*	0.04	1.4
202430_s_at	*PLSCR1*	0.04	1.4
228617_at	*XAF1*	0.04	1.4
209047_at	*AQP1*	0.05	1.4
215554_at	*GPLD1*	0.01	1.39

232750_at		0.01	1.39
215073_s_at	*NR2F2*	0.02	1.39
231848_x_at	*ZNF207*	0.02	1.39
225598_at	*SLC45A4*	0.02	1.39
205756_s_at	*F8*	0.02	1.39
241689_at	*METTL14*	0.03	1.39
216713_at	*KRIT1*	0.03	1.39
1560048_at		0.03	1.39
1560661_x_at	*LINC00342*	0.03	1.39
218723_s_at	*RGCC*	0.04	1.39
212822_at	*HEG1*	0.04	1.39
214022_s_at	*IFITM1*	0.04	1.39
201140_s_at	*RAB5C*	0.04	1.39
201939_at	*PLK2*	0.04	1.39
1566887_x_at		0.05	1.39
220116_at	*KCNN2*	0.05	1.39
200789_at	*ECH1*	0.00	1.38
217317_s_at	*HERC2P2///HERC2P9///LOC105369242*	0.01	1.38
204624_at	*ATP7B*	0.01	1.38
210605_s_at	*MFGE8*	0.01	1.38
60528_at	*JMJD7-PLA2G4B///PLA2G4B*	0.01	1.38
227012_at	*SLC25A40*	0.02	1.38
212080_at	*KMT2A*	0.02	1.38
219647_at	*POPDC2*	0.03	1.38
226363_at	*ABCC5*	0.03	1.38
242131_at	*ATP6*	0.03	1.38
224441_s_at	*USP45*	0.04	1.38
224658_x_at	*PACS1*	0.05	1.38
240155_x_at	*ZNF493*	0.01	1.37
236431_at	*U2SURP*	0.01	1.37
239849_at		0.01	1.37
205848_at	*GAS2*	0.01	1.37
213204_at	*CUL9*	0.02	1.37
234297_at	*RGS8///SDHAP3*	0.02	1.37
59631_at	*TXNRD3///TXNRD3NB*	0.02	1.37
229861_at	*LIG3*	0.02	1.37
240467_at	*LOC105373341*	0.02	1.37
218868_at	*ACTR3B*	0.03	1.37
235088_at	*C4orf46*	0.05	1.37
228239_at	*SMIM11A///SMIM11B*	0.01	1.36
212088_at	*PMPCA*	0.01	1.36
225947_at	*MYO19*	0.01	1.36
225968_at	*PRICKLE2*	0.01	1.36
213213_at	*DIDO1*	0.02	1.36
204067_at	*SUOX*	0.02	1.36
219134_at	*ADGRL4*	0.02	1.36
238458_at	*MICU3*	0.02	1.36
209196_at	*WDR46*	0.03	1.36
231947_at	*MYCT1*	0.01	1.35
224821_at	*ABHD14B*	0.02	1.35
38269_at	*PRKD2*	0.02	1.35
219378_at	*NAA16*	0.02	1.35
214369_s_at	*RASGRP2*	0.03	1.35
202234_s_at	*SLC16A1*	0.03	1.35

207598_x_at	*XRCC2*	0.03	1.35
1558097_at	*PRR14L*	0.04	1.35
217144_at		0.04	1.35
203657_s_at	*CTSF*	0.04	1.35
227449_at	*EPHA4*	0.04	1.35
208690_s_at	*PDLIM1*	0.01	1.34
209605_at	*TST*	0.01	1.34
232852_at		0.02	1.34
215483_at	*AKAP9*	0.02	1.34
236283_x_at	*LOC646214*	0.02	1.34
218381_s_at	*U2AF2*	0.02	1.34
226259_at	*EXOC6*	0.02	1.34
203920_at	*NR1H3*	0.02	1.34
224719_s_at	*C12orf57*	0.02	1.34
244209_at	*LBX1-AS1*	0.03	1.34
203839_s_at	*TNK2*	0.04	1.34
203122_at	*TRAPPC12*	0.04	1.34
214934_at	*ATP9B*	0.04	1.34
238199_x_at	*COX3*	0.04	1.34
213183_s_at		0.05	1.34
203323_at	*CAV2*	0.01	1.33
219440_at	*RAI2*	0.02	1.33
231991_at	*CCM2L*	0.02	1.33
230256_at	*RUSC1-AS1*	0.02	1.33
212947_at	*SLC9A8*	0.02	1.33
1552318_at	*GIMAP1*	0.02	1.33
218124_at	*RETSAT*	0.03	1.33
240798_at		0.04	1.33
203295_s_at	*ATP1A2*	0.04	1.33
238653_at	*LOC102723919///LRIG2*	0.05	1.33
239423_at		0.05	1.33
215604_x_at		0.05	1.33
226356_at	*FAM73B*	0.01	1.32
204635_at	*RPS6KA5*	0.01	1.32
244398_x_at	*ZNF684*	0.02	1.32
220113_x_at	*POLR1B*	0.02	1.32
215766_at	*GSTA5*	0.02	1.32
228084_at	*CASP6///PLA2G12A*	0.02	1.32
202071_at	*SDC4*	0.02	1.32
52159_at	*HEMK1*	0.02	1.32
222603_at	*ERMP1*	0.02	1.32
40225_at	*GAK*	0.03	1.32
225293_at	*COL27A1*	0.03	1.32
227502_at		0.03	1.32
223192_at	*SLC25A28*	0.03	1.32
213215_at	*AP3S2///C15orf38-AP3S2*	0.03	1.32
237189_at	*HOXB-AS1*	0.03	1.32
243271_at		0.03	1.32
213046_at	*PABPN1*	0.03	1.32
204464_s_at	*EDNRA*	0.04	1.32
202112_at	*VWF*	0.04	1.32
209512_at	*HSDL2*	0.04	1.32
242849_at		0.04	1.32
1553218_a_at	*ZNF512*	0.04	1.32

221589_s_at	*ALDH6A1*	0.05	1.32
204294_at	*AMT*	0.05	1.32
202812_at	*GAA*	0.01	1.31
231940_at	*ZNF529*	0.02	1.31
201005_at	*CD9*	0.02	1.31
202943_s_at	*NAGA*	0.03	1.31
219680_at	*NLRX1*	0.03	1.31
231839_at	*PDE12*	0.03	1.31
218706_s_at	*GRAMD3*	0.04	1.31
205589_at	*MYL3*	0.04	1.31
223698_at	*SLC25A36*	0.04	1.31
242974_at		0.04	1.31
209306_s_at	*SWAP70*	0.04	1.31
1557539_at		0.05	1.31
236514_at	*ACOT8*	0.05	1.31
201578_at	*PODXL*	0.05	1.31
212928_at	*TSPYL4*	0.02	1.3
214268_s_at	*MTMR4*	0.02	1.3
208922_s_at	*NXF1*	0.03	1.3
228482_at	*CDRT4///TVP23C-CDRT4*	0.03	1.3
208309_s_at	*MALT1*	0.04	1.3
220221_at	*VPS13D*	0.04	1.3
205773_at	*CPEB3*	0.05	1.3

Supplementary Table S2. Downregulated genes differentially expressed in athletes ($p < 0.05$ and -1.3–fold change)

	Gene Symbol	*p* Value	Fold Change
205960_at	*PDK4*	0.01	-2.68
224568_x_at	*MALAT1*	0.01	-2.64
205018_s_at	*MBNL2*	0.01	-2.59
217097_s_at	*PHTF2*	0.02	-2.3
1558924_s_at	*CLIP1*	0.01	-2.27
1558028_x_at	*LINC00657*	0.00	-2.24
206765_at	*KCNJ2*	0.00	-2.21
235216_at	*ESCO1*	0.03	-2.2
211022_s_at	*ATRX*	0.01	-2.14
212257_s_at	*SMARCA2*	0.01	-2.12
209055_s_at	*CDC5L*	0.01	-2.11
1569472_s_at	*TTC3*	0.03	-2.11
	1557987_at	0.00	-2.1
223940_x_at	*MALAT1*	0.02	-2.07
1555567_s_at	*LMOD3*	0.01	-2.05
1557227_s_at	*TPR*	0.03	-2.05
211993_at	*WNK1*	0.04	-2.05
	AFFX-HUMRGE /M10098_M_at	0.00	-2.04
217576_x_at	*SOS2*	0.01	-2.04
1557910_at	*HSP90AB1*	0.03	-2.03
241955_at	*HECTD1*	0.04	-2
235312_s_at	*NRAP*	0.02	-1.99
211000_s_at	*IL6ST*	0.03	-1.98
204840_s_at	*EEA1*	0.04	-1.98
1554249_a_at	*ZNF638*	0.01	-1.95
204324_s_at	*GOLIM4*	0.04	-1.95
209895_at	*PTPN11*	0.01	-1.93
201008_s_at	*TXNIP*	0.01	-1.91
217644_s_at	*SOS2*	0.02	-1.89
	AFFX-HUMRGE /M10098_5_at	0.01	-1.86
212272_at	*LPIN1*	0.01	-1.83
236374_at	*CTXN3*	0.04	-1.83
213470_s_at	*HNRNPH1*	0.00	-1.82
223704_s_at	*DMRT2*	0.01	-1.82
204863_s_at	*IL6ST*	0.02	-1.8
241683_at	*HECTD1*	0.01	-1.79
232704_s_at	*LRRFIP2*	0.02	-1.79
208297_s_at	*EVI5*	0.05	-1.79
219321_at	*MPP5*	0.01	-1.77
243855_at		0.01	-1.76
1555167_s_at	*NAMPT*	0.02	-1.75
1555594_a_at	*MBNL1*	0.01	-1.74
212570_at	*ENDOD1*	0.04	-1.73
215505_s_at	*STRN3*	0.00	-1.72
212720_at	*PAPOLA*	0.01	-1.72
1558015_s_at	*ACTR2*	0.05	-1.72
211169_s_at	*PPP1R3A*	0.00	-1.71
243648_at	*ZBED6*	0.01	-1.71

1555436_a_at	*AFF4*	0.01	-1.71
202118_s_at	*CPNE3*	0.04	-1.71
221487_s_at	*ENSA*	0.04	-1.71
209896_s_at	*PTPN11*	0.01	-1.69
238146_at		0.02	-1.69
231513_at		0.02	-1.69
209203_s_at	*BICD2*	0.04	-1.69
242280_x_at	*CPEB4*	0.02	-1.68
235295_at	*PANX1*	0.05	-1.68
215191_at		0.00	-1.67
209023_s_at	*STAG2*	0.01	-1.67
224828_at	*CPEB4*	0.01	-1.67
207968_s_at	*MEF2C*	0.03	-1.67
236557_at	*ZBTB38*	0.04	-1.67
201946_s_at	*CCT2*	0.00	-1.66
242279_at		0.00	-1.66
211937_at	*EIF4B*	0.01	-1.66
1561004_at	*PRKCQ-AS1*	0.01	-1.66
1565823_at		0.02	-1.66
202464_s_at	*PFKFB3*	0.04	-1.66
238341_at		0.05	-1.66
1553122_s_at	*RBAK*	0.05	-1.66
207977_s_at	*DPT*	0.02	-1.65
201635_s_at	*FXR1*	0.02	-1.65
212420_at	*ELF1*	0.03	-1.65
	AFFX-ThrX-5_at	0.02	-1.64
212650_at	*EHBP1*	0.03	-1.64
203851_at	*IGFBP6*	0.04	-1.64
200806_s_at	*HSPD1*	0.01	-1.63
201337_s_at	*VAMP3*	0.04	-1.63
202558_s_at	*HSPA13*	0.01	-1.62
201101_s_at	*BCLAF1*	0.01	-1.62
211968_s_at	*HSP90AA1*	0.01	-1.62
212587_s_at	*PTPRC*	0.03	-1.62
221094_s_at	*ELP3*	0.03	-1.62
209676_at	*TFPI*	0.01	-1.61
215236_s_at	*PICALM*	0.04	-1.61
214085_x_at	*GLIPR1*	0.01	-1.6
213554_s_at	*CDV3*	0.03	-1.6
242732_at		0.03	-1.6
214544_s_at	*SNAP23*	0.03	-1.6
220467_at		0.04	-1.6
242088_at	*KLHL24*	0.04	-1.6
219679_s_at	*WAC*	0.00	-1.59
202619_s_at	*PLOD2*	0.01	-1.59
207983_s_at	*STAG2*	0.01	-1.59
235765_at	*TLE4*	0.02	-1.59
1555814_a_at	*RHOA*	0.02	-1.59
205809_s_at	*WASL*	0.05	-1.59
243552_at	*MBTD1*	0.01	-1.58
201399_s_at	*TRAM1*	0.01	-1.58
204969_s_at	*RDX*	0.02	-1.58
224566_at	*NEAT1*	0.02	-1.58
216449_x_at	*HSP90B1*	0.03	-1.58

1554807_a_at	*SPIRE1*	0.03	-1.58
215434_x_at	*LOC102724250///NBPF1///NBPF10///NBPF19*	0.03	-1.58
213875_x_at	*C6orf62*	0.05	-1.58
233819_s_at	*LTN1*	0.00	-1.57
208653_s_at	*CD164*	0.02	-1.57
227223_at	*RBM39*	0.02	-1.57
209997_x_at	*PCM1*	0.02	-1.57
238960_s_at	*LARP4*	0.03	-1.57
223701_s_at	*USP47*	0.03	-1.57
203377_s_at	*CDC40*	0.03	-1.57
206891_at	*ACTN3*	0.04	-1.57
214352_s_at	*KRAS*	0.04	-1.57
225018_at	*SPIRE1*	0.05	-1.57
217951_s_at	*PHF3*	0.01	-1.56
223904_at	*PRKAG3*	0.01	-1.56
1555193_a_at	*ZNF277*	0.01	-1.56
1554717_a_at	*PDE4D*	0.03	-1.56
1558093_s_at	*MATR3*	0.04	-1.56
242352_at	*NIPBL*	0.04	-1.56
240601_at		0.01	-1.55
1554014_at	*CHD2*	0.03	-1.55
228523_at	*NANOS1*	0.04	-1.55
201514_s_at	*G3BP1*	0.04	-1.55
	AFFX-HUMRGE /M10098_3_at	0.01	-1.54
225119_at	*CHMP4B*	0.01	-1.54
222725_s_at	*PALMD*	0.02	-1.54
217966_s_at	*FAM129A*	0.02	-1.54
1569110_x_at	*LOC728613*	0.04	-1.54
204507_s_at	*PPP3R1*	0.02	-1.53
233208_x_at	*CPSF2*	0.04	-1.53
215330_at		0.01	-1.52
222834_s_at	*GNG12*	0.02	-1.52
226404_at	*RBM39*	0.04	-1.52
33148_at	*ZFR*	0.04	-1.52
239771_at	*CAND1*	0.01	-1.51
225863_s_at	*C19orf12*	0.02	-1.51
206373_at	*ZIC1*	0.02	-1.51
235180_at	*STYX*	0.03	-1.51
243861_at	*FNIP1*	0.04	-1.51
207549_x_at	*CD46*	0.04	-1.51
238792_at	*PCNX*	0.04	-1.51
215233_at	*JMJD6*	0.01	-1.5
215263_at	*ZXDA///ZXDB*	0.01	-1.5
211578_s_at	*RPS6KB1*	0.01	-1.5
206050_s_at	*RNH1*	0.03	-1.5
240602_at	*HBS1L*	0.04	-1.5
230130_at	*SLIT2*	0.05	-1.5
203811_s_at	*DNAJB4*	0.01	-1.49
233230_s_at	*SLAIN2*	0.02	-1.49
223254_s_at	*G2E3*	0.03	-1.49
224642_at	*FYTTD1*	0.04	-1.49
218521_s_at	*UBE2W*	0.04	-1.49
213286_at	*ZFR*	0.05	-1.49

202547_s_at	*ARHGEF7*	0.00	-1.48
210664_s_at	*TFPI*	0.01	-1.48
202290_at	*PDAP1*	0.02	-1.48
212106_at	*FAF2*	0.02	-1.48
224046_s_at	*PDE7A*	0.02	-1.48
216125_s_at	*RANBP9*	0.03	-1.48
242467_at		0.03	-1.48
233799_at		0.01	-1.47
214305_s_at	*SF3B1*	0.01	-1.47
206809_s_at	*HNRNPA3///HNRNPA3P1*	0.02	-1.47
203579_s_at	*SLC7A6*	0.02	-1.47
224601_at	*SNHG16*	0.02	-1.47
237721_s_at	*ASB4*	0.02	-1.47
204782_at		0.04	-1.47
212286_at	*ANKRD12*	0.04	-1.47
1555892_s_at	*PSMD5-AS1*	0.04	-1.47
201298_s_at	*MOB1A*	0.01	-1.46
223138_s_at	*DHX36*	0.01	-1.46
1555618_s_at	*SAE1*	0.01	-1.46
212758_s_at	*ZEB1*	0.01	-1.46
203425_s_at	*IGFBP5*	0.01	-1.46
235484_at	*PTAR1*	0.02	-1.46
222529_at	*SLC25A37*	0.02	-1.46
212093_s_at	*MTUS1*	0.03	-1.46
212398_at	*RDX*	0.04	-1.46
238455_at	*PLXDC2*	0.04	-1.46
206876_at	*SIM1*	0.04	-1.46
235440_at	*SPTY2D1*	0.01	-1.45
226315_at	*ZNF830*	0.01	-1.45
204340_at	*TMEM187*	0.01	-1.45
229606_at		0.01	-1.45
216521_s_at	*BRCC3*	0.02	-1.45
218218_at	*APPL2*	0.02	-1.45
234975_at	*GSPT1*	0.03	-1.45
212404_s_at	*UBE3B*	0.04	-1.45
239504_at		0.05	-1.45
223996_s_at	*MRPL30*	0.00	-1.44
219380_x_at	*POLH*	0.01	-1.44
224472_x_at	*SDF4*	0.01	-1.44
209808_x_at	*ING1*	0.02	-1.44
223879_s_at	*OXR1*	0.03	-1.44
217598_at	*CINP*	0.03	-1.44
210251_s_at	*RUFY3*	0.04	-1.44
209846_s_at	*BTN3A2*	0.05	-1.44
219600_s_at	*TMEM50B*	0.05	-1.44
212641_at	*HIVEP2*	0.01	-1.43
202412_s_at	*USP1*	0.01	-1.43
244783_at	*YAF2*	0.02	-1.43
220368_s_at	*PPP4R3A*	0.02	-1.43
208840_s_at	*G3BP2*	0.03	-1.43
226090_x_at	*RABL3*	0.03	-1.43
216008_s_at	*ARIH2*	0.00	-1.42
217356_s_at	*PGK1*	0.01	-1.42
212514_x_at	*DDX3X*	0.01	-1.42

223016_x_at	*ZRANB2*	0.01	-1.42
225055_at	*LINC00674*	0.01	-1.42
225438_at	*NUDCD1*	0.02	-1.42
201983_s_at	*EGFR*	0.02	-1.42
204427_s_at	*TMED2*	0.02	-1.42
236354_at		0.03	-1.42
201372_s_at	*CUL3*	0.03	-1.42
204577_s_at	*CLUAP1*	0.03	-1.42
216100_s_at	*TOR1AIP1*	0.03	-1.42
218006_s_at	*ZNF22*	0.05	-1.42
203491_s_at	*CEP57*	0.02	-1.41
232344_at		0.02	-1.41
202915_s_at	*FAM20B*	0.02	-1.41
213473_at	*BRAP*	0.03	-1.41
235730_at	*NUTM2B-AS1*	0.03	-1.41
228315_at	*ZMAT3*	0.03	-1.41
206704_at	*CLCN5*	0.03	-1.41
221571_at	*TRAF3*	0.04	-1.41
1554062_at	*XG*	0.04	-1.41
200900_s_at	*M6PR*	0.00	-1.4
221740_x_at	*LRRC37A2*	0.01	-1.4
236388_at	*STRN*	0.01	-1.4
228561_at	*CDC37L1*	0.01	-1.4
209257_s_at	*SMC3*	0.01	-1.4
224661_at	*PIGY///PYURF*	0.02	-1.4
219250_s_at	*FLRT3*	0.02	-1.4
212246_at	*MCFD2*	0.02	-1.4
219908_at	*DKK2*	0.02	-1.4
214121_x_at	*PDLIM7*	0.03	-1.4
243835_at	*ZDHHC21*	0.04	-1.4
204066_s_at	*AGAP1*	0.05	-1.4
206652_at	*ZMYM5*	0.05	-1.4
1554577_a_at	*PSMD10*	0.00	-1.39
208447_s_at	*PRPS1*	0.01	-1.39
211090_s_at	*PRPF4B*	0.03	-1.39
219198_at	*GTF3C4*	0.04	-1.39
229193_at	*LUC7L3*	0.04	-1.39
1558111_at	*MBNL1*	0.04	-1.39
238787_at	*DENND1B*	0.01	-1.38
212073_at	*CSNK2A1*	0.02	-1.38
235388_at	*CHD9*	0.02	-1.38
243904_at	*STXBP5*	0.02	-1.38
234980_at	*TMEM56*	0.02	-1.38
240310_at	*TOR1AIP1*	0.03	-1.38
1555945_s_at	*FAM120A*	0.04	-1.38
230904_at	*FSD1L*	0.04	-1.38
236524_at		0.05	-1.38
208765_s_at	*HNRNPR*	0.02	-1.37
211387_x_at	*RNGTT*	0.03	-1.37
203437_at	*TMEM11*	0.03	-1.37
212595_s_at	*DAZAP2*	0.04	-1.37
200889_s_at	*SSR1*	0.05	-1.37
241403_at	*CLK4*	0.05	-1.37
203530_s_at	*STX4*	0.01	-1.36

201606_s_at	*PWP1*	0.01	-1.36
225402_at	*TP53RK*	0.01	-1.36
214869_x_at	*GAPVD1*	0.02	-1.36
224576_at	*ERGIC1*	0.02	-1.36
1554863_s_at	*DOK5*	0.04	-1.36
228495_at	*GPATCH11*	0.04	-1.36
218573_at	*MAGEH1*	0.04	-1.36
225956_at	*CREBRF*	0.04	-1.36
208875_s_at	*PAK2*	0.02	-1.35
1563111_a_at	*PIGX*	0.02	-1.35
200751_s_at	*HNRNPC*	0.02	-1.35
209862_s_at	*CEP57*	0.02	-1.35
243963_at		0.03	-1.35
227229_at	*VPS53*	0.04	-1.35
222120_at	*ZNF764*	0.04	-1.35
230057_at	*LOC285178*	0.04	-1.35
244804_at	*SQSTM1*	0.04	-1.35
215024_at	*CCZ1B*	0.05	-1.35
1553366_s_at	*ANKRD23///ANKRD39*	0.05	-1.35
212932_at	*RAB3GAP1*	0.05	-1.35
214783_s_at	*ANXA11*	0.01	-1.34
1554127_s_at	*MSRB3*	0.02	-1.34
201538_s_at	*DUSP3*	0.03	-1.34
224851_at	*CDK6*	0.03	-1.34
215707_s_at	*PRNP*	0.04	-1.34
201761_at	*MTHFD2*	0.05	-1.34
235344_at	*PPM1A*	0.05	-1.34
201457_x_at	*BUB3*	0.01	-1.33
1560609_at	*CRYZL1*	0.01	-1.33
202089_s_at	*SLC39A6*	0.01	-1.33
212196_at	*IL6ST*	0.02	-1.33
204185_x_at	*PPID*	0.02	-1.33
201723_s_at	*GALNT1*	0.02	-1.33
225546_at	*EEF2K///LOC101930123*	0.03	-1.33
214820_at	*BRWD1*	0.03	-1.33
213805_at	*ABHD5*	0.03	-1.33
202292_x_at	*LYPLA2*	0.03	-1.33
235787_at	*CDC37L1*	0.04	-1.33
200959_at	*FUS*	0.01	-1.32
209744_x_at	*ITCH*	0.02	-1.32
201988_s_at	*CREBL2*	0.02	-1.32
242586_at	*FSD1L*	0.03	-1.32
216640_s_at	*PDIA6*	0.03	-1.32
208945_s_at	*BECN1*	0.03	-1.32
235190_at		0.03	-1.32
	1565886_at	0.03	-1.32
225896_at	*MPRIP*	0.04	-1.32
224797_at	*ARRDC3*	0.04	-1.32
209726_at	*CA11*	0.05	-1.32
221088_s_at	*PPP1R9A*	0.05	-1.32
1557167_at	*HCG11*	0.02	-1.31
208696_at	*CCT5*	0.02	-1.31
218539_at	*FBXO34*	0.03	-1.31
223527_s_at	*CDADC1*	0.03	-1.31

214545_s_at	*PROSC*	0.03	-1.31
215109_at	*RC3H1*	0.03	-1.31
205543_at	*HSPA4L*	0.04	-1.31
201593_s_at	*ZC3H15*	0.01	-1.3
203622_s_at	*PNO1*	0.01	-1.3
201201_at	*CSTB*	0.01	-1.3
217850_at	*GNL3*	0.01	-1.3
200001_at	*CAPNS1*	0.02	-1.3
201774_s_at	*NCAPD2*	0.03	-1.3
224905_at	*WDR26*	0.03	-1.3
203273_s_at	*TUSC2*	0.03	-1.3
37232_at	*KIAA0586*	0.04	-1.3
1553148_a_at	*SNX13*	0.04	-1.3

Supplementary Table S3. Significantly upregulated Gene Ontology gene sets in the control group

	Name	Size	NOM *p*-value	FDR q-value
1	GO_RNA_BINDING	61	0.00E+00	2.53E-03
2	GO_RIBONUCLEOPROTEIN_COMPLEX	30	0.00E+00	7.59E-03
3	GO_PROTEIN_CONTAINING_COMPLEX_ASSEMBLY	60	0.00E+00	1.97E-02
4	GO_RESPONSE_TO_ABIOTIC_STIMULUS	47	0.00E+00	2.77E-02
5	GO_CHROMOSOME	33	0.00E+00	2.39E-02
6	GO_CELLULAR_PROTEIN_CONTAINING_COMPLEX_ASSEMBLY	37	0.00E+00	3.22E-02
7	GO_CELLULAR_PROTEIN_CATABOLIC_PROCESS	27	0.00E+00	4.86E-02
8	GO_MRNA_BINDING	17	0.00E+00	4.92E-02
9	GO_ENZYME_REGULATOR_ACTIVITY	34	0.00E+00	5.17E-02
10	GO_CELLULAR_MACROMOLECULE_CATABOLIC_PROCESS	38	1.70E-03	5.05E-02
11	GO_PROTEIN_CONTAINING_COMPLEX_BINDING	39	1.73E-03	5.64E-02
12	GO_RNA_SPLICING	24	1.78E-03	4.00E-02
13	GO_REGULATION_OF_RESPONSE_TO_STRESS	44	1.79E-03	4.91E-02
14	GO_CHROMATIN_BINDING	16	1.83E-03	5.22E-02
15	GO_CATALYTIC_COMPLEX	41	1.84E-03	4.54E-02
16	GO_POSITIVE_REGULATION_OF_CELL_DEATH	18	1.98E-03	4.69E-02
17	GO_MODIFICATION_DEPENDENT_MACROMOLECULE_CATABOLIC_PROCESS	22	3.45E-03	4.04E-02
18	GO_NEGATIVE_REGULATION_OF_BIOSYNTHETIC_PROCESS	49	3.53E-03	5.57E-02
19	GO_CHROMOSOME_ORGANIZATION	41	3.57E-03	3.17E-02
20	GO_REGULATION_OF_NUCLEOBASE_CONTAINING_COMPOUND_METABOLIC_PROCESS	25	3.57E-03	7.70E-02
21	GO_MRNA_METABOLIC_PROCESS	27	3.66E-03	5.50E-02
22	GO_NEGATIVE_REGULATION_OF_RESPONSE_TO_STIMULUS	46	5.33E-03	1.10E-01
23	GO_CELLULAR_RESPONSE_TO_DNA_DAMAGE_STIMULUS	27	5.35E-03	5.36E-02
24	GO_REGULATION_OF_CELL_DEATH	48	5.40E-03	8.10E-02
25	GO_REGULATION_OF_CELLULAR_RESPONSE_TO_STRESS	25	5.42E-03	7.86E-02
26	GO_RNA_SPLICING_VIA_TRANSESTERIFICATION_REACTIONS	19	6.80E-03	7.00E-02
27	GO_CHROMATIN_ORGANIZATION	21	7.21E-03	7.88E-02
28	GO_RNA_METABOLIC_PROCESS	35	7.30E-03	8.07E-02
29	GO_DNA_METABOLIC_PROCESS	32	8.65E-03	1.01E-01
30	GO_PROTEIN_MODIFICATION_BY_SMALL_PROTEIN_CONJUGATION_OR_REMOVAL	34	8.88E-03	8.21E-02

31	GO_NUCLEAR_SPECK	21	8.91E-03	8.14E-02
32	GO_ESTABLISHMENT_OF_PROTEIN_LOCALIZATION_TO_ORGANELLE	25	8.93E-03	7.46E-02
33	GO_PROTEIN_MODIFICATION_BY_SMALL_PROTEIN_CONJUGATION	27	9.33E-03	1.10E-01
34	GO_REGULATION_OF_DNA_METABOLIC_PROCESS	16	9.45E-03	1.11E-01
35	GO_CELLULAR_MACROMOLECULE_LOCALIZATION	76	1.02E-02	1.11E-01
36	GO_CELL_SURFACE_RECEPTOR_SIGNALING_PATHWAY_INVOLVED_IN_CELL_CELL_SIGNALI NG	21	1.03E-02	7.42E-02
37	GO_MACROMOLECULE_CATABOLIC_PROCESS	45	1.05E-02	1.15E-01
38	GO_NUCLEAR_BODY	27	1.07E-02	1.10E-01
39	GO_T_CELL_ACTIVATION	16	1.26E-02	1.18E-01
40	GO_ATPASE_ACTIVITY	17	1.30E-02	1.28E-01
41	GO_NUCLEOPLASM_PART	33	1.39E-02	9.03E-02
42	GO_REGULATION_OF_CELLULAR_AMIDE_METABOLIC_PROCESS	20	1.41E-02	1.33E-01
43	GO_PROTEIN_CATABOLIC_PROCESS	31	1.45E-02	8.97E-02
44	GO_NEGATIVE_REGULATION_OF_CELL_CYCLE	27	1.74E-02	7.47E-02
45	GO_NEURON_PART	53	1.75E-02	1.33E-01
46	GO_REGULATION_OF_INTRACELLULAR_SIGNAL_TRANSDUCTION	47	1.77E-02	1.25E-01
47	GO_REGULATION_OF_GROWTH	22	1.80E-02	1.25E-01
48	GO_APOPTOTIC_PROCESS	54	1.81E-02	1.12E-01
49	GO_CELL_GROWTH	18	1.96E-02	1.28E-01
50	GO_PROTEIN_DOMAIN_SPECIFIC_BINDING	34	2.01E-02	1.10E-01
51	GO_MICROTUBULE_CYTOSKELETON_ORGANIZATION	20	2.05E-02	1.20E-01
52	GO_PEPTIDE_BIOSYNTHETIC_PROCESS	22	2.09E-02	1.59E-01
53	GO_CELL_CYCLE	71	2.10E-02	1.27E-01
54	GO_CELL_CELL_SIGNALING_BY_WNT	18	2.19E-02	1.15E-01
55	GO_NEGATIVE_REGULATION_OF_INTRACELLULAR_SIGNAL_TRANSDUCTION	17	2.40E-02	1.25E-01
56	GO_PROTEIN_LOCALIZATION_TO_ORGANELLE	39	2.48E-02	1.35E-01
57	GO_NEGATIVE_REGULATION_OF_CELL_CYCLE_PROCESS	16	2.55E-02	1.44E-01
58	GO_CHROMOSOME_SEGREGATION	16	2.67E-02	1.39E-01
59	GO_POSTTRANSCRIPTIONAL_REGULATION_OF_GENE_EXPRESSION	24	2.68E-02	1.25E-01
60	GO_REGULATION_OF_CELL_CYCLE	46	3.10E-02	1.67E-01
61	GO_CYTOSKELETAL_PROTEIN_BINDING	38	3.22E-02	1.98E-01
62	GO_GROWTH	35	3.29E-02	1.38E-01

63	GO_PROTEOLYSIS	48	3.74E-02	1.58E-01
64	GO_DNA_REPAIR	19	3.79E-02	1.58E-01
65	GO_NEGATIVE_REGULATION_OF_SIGNALING	41	3.81E-02	1.97E-01
66	GO_RESPONSE_TO_RADIATION	15	3.95E-02	1.70E-01
67	GO_SYNAPSE_PART	32	3.99E-02	1.67E-01
68	GO_PROTEIN_DIMERIZATION_ACTIVITY	46	4.62E-02	2.05E-01
69	GO_REGULATION_OF_PROTEOLYSIS	23	4.83E-02	2.17E-01
70	GO_NEGATIVE_REGULATION_OF_PROTEIN_METABOLIC_PROCESS	43	4.90E-02	2.08E-01

NOM, Nominal; FDR, False discovery rate.

Supplementary Table S4. Significantly upregulated Gene Ontology gene sets in the athlete group

	Name	Size	NOM *p*-value	FDR q-value
1	GO_BLOOD_VESSEL_MORPHOGENESIS	23	0.00E+00	3.37E-01
2	GO_ANATOMICAL_STRUCTURE_FORMATION_INVOLVED_IN_MORPHOGENESIS	35	4.68E-03	2.38E-01
3	GO_CARDIOVASCULAR_SYSTEM_DEVELOPMENT	25	6.62E-03	3.11E-01
4	GO_TRANSPORTER_ACTIVITY	29	9.48E-03	4.05E-01
5	GO_REGULATION_OF_HORMONE_LEVELS	15	1.28E-02	2.68E-01
6	GO_CIRCULATORY_SYSTEM_DEVELOPMENT	38	1.31E-02	2.68E-01
7	GO_TUBE_DEVELOPMENT	39	1.63E-02	2.80E-01
8	GO_TISSUE_MIGRATION	15	1.79E-02	2.39E-01
9	GO_CIRCULATORY_SYSTEM_PROCESS	26	2.27E-02	2.44E-01
10	GO_TRANSMEMBRANE_TRANSPORTER_ACTIVITY	25	2.40E-02	2.47E-01
11	GO_SMALL_MOLECULE_METABOLIC_PROCESS	47	4.41E-02	3.90E-01
12	GO_RESPONSE_TO_LIPID	26	4.51E-02	4.53E-01
13	GO_HEART_PROCESS	15	4.82E-02	4.75E-01

NOM, Nominal; FDR, False discovery rate.

Supplementary Table S5. Significantly upregulated Reactome Pathway gene sets in the control group

	Name	Size	NOM *p*-value	FDR q-value
1	REACTOME_CELL_CYCLE	23	0.00E+00	2.17E-02
2	REACTOME_INNATE_IMMUNE_SYSTEM	39	1.75E-03	2.83E-02
3	REACTOME_CELL_CYCLE_MITOTIC	21	5.58E-03	1.75E-02
4	REACTOME_AXON_GUIDANCE	28	7.27E-03	2.88E-02
5	REACTOME_M_PHASE	17	9.19E-03	2.72E-02
6	REACTOME_SIGNALING_BY_RECEPTOR_TYROSINE_KINASES	16	2.76E-02	8.76E-02
7	REACTOME_DEVELOPMENTAL_BIOLOGY	35	3.48E-02	9.93E-02

NOM, Nominal; FDR, False discovery rate.

Supplementary Table S6. Gene expression changes in "RESPONSE TO LIPID"

Gene Symbol	Description	Rank in Gene List	Rank Metric Score	Running es	Core Enrichment	*p* Value	Fold Change
STRN3	striatin 3	0	1.28	0.06	No	0.00	-1.72
TXNIP	thioredoxin interacting protein	52	0.87	0.00	No	0.01	-1.91
TFPI	tissue factor pathway inhibitor	56	0.86	0.03	No	0.01	-1.61
GNG12	G protein subunit gamma 12	85	0.82	0.01	No	0.02	-1.52
MEF2C	myocyte enhancer factor 2C	100	0.79	0.02	No	0.03	-1.67
KRAS	KRAS proto-oncogene, GTPase	104	0.79	0.05	No	0.04	-1.57
RHOA	ras homolog family member A	204	0.70	-0.12	No	0.02	-1.59
PDE4D	phosphodiesterase 4D	236	0.67	-0.15	No	0.03	-1.56
EGFR	epidermal growth factor receptor	237	0.67	-0.12	No	0.02	-1.42
CREBRF	CREB3 regulatory factor	245	0.65	-0.10	No	0.04	-1.36
PDK4	pyruvate dehydrogenase kinase 4	274	0.58	-0.13	No	0.01	-2.68
ATP1A2	ATPase Na+/K+ transporting subunit alpha 2	289	-0.58	-0.13	No	0.04	1.33
AQP1	aquaporin 1 (Colton blood group)	297	-0.62	-0.12	No	0.05	1.40
PABPN1	poly(A) binding protein nuclear 1	300	-0.63	-0.09	No	0.03	1.32
MALT1	MALT1 paracaspase	331	-0.66	-0.12	No	0.04	1.30
NR1H3	nuclear receptor subfamily 1 group H member 3	384	-0.71	-0.20	No	0.02	1.34
ESRRG	estrogen related receptor gamma	419	-0.75	-0.23	No	0.03	1.40
NR2F2	nuclear receptor subfamily 2 group F member 2	442	-0.77	-0.24	Yes	0.02	1.39
IRAK2	interleukin 1 receptor associated kinase 2	453	-0.79	-0.22	Yes	0.03	1.58
ESRRB	estrogen related receptor beta	468	-0.82	-0.21	Yes	0.01	1.52
SCARB1	scavenger receptor class B member 1	481	-0.86	-0.20	Yes	0.02	1.59
GPLD1	glycosylphosphatidylinositol specific phospholipase D1	483	-0.87	-0.16	Yes	0.02	1.54
KCNJ8	potassium voltage-gated channel subfamily J member 8	501	-1.00	-0.14	Yes	0.00	1.48
EDN1	endothelin 1	502	-1.01	-0.10	Yes	0.01	1.53
GATA2	GATA binding protein 2	503	-1.01	-0.05	Yes	0.00	1.51
ASS1	argininosuccinate synthase 1	510	-1.26	0.00	Yes	0.00	1.85
